# Bioactive Phenolic Compounds in the Modulation of Central and Peripheral Nervous System Cancers: Facts and Misdeeds

**DOI:** 10.3390/cancers12020454

**Published:** 2020-02-15

**Authors:** Lorena Perrone, Simone Sampaolo, Mariarosa Anna Beatrice Melone

**Affiliations:** 1 Department of Advanced Medical and Surgical Sciences, 2nd Division of Neurology, Center for Rare Diseases and InterUniversity Center for Research in Neurosciences, University of Campania “Luigi Vanvitelli”, Via Sergio Pansini, 5 80131 Naples, Italy; perronelorena1@gmail.com (L.P.); simone.sampaolo@unicampania.it (S.S.); 2 Department of Chemistry and Biology, University Grenoble Alpes, 38400 Saint-Martin-d’Hères, France; 3 Sbarro Institute for Cancer Research and Molecular Medicine, Department of Biology, Temple University, BioLife Building (015-00)1900 North 12th Street, Philadelphia, PA 19122-6078, USA

**Keywords:** brain cancer, gliomas, schwannomas, malignant tumors of the peripheral nerve sheath (MPNST), neurofibromas, polyphenols, bioavailability, nanoparticle-based delivery systems

## Abstract

Efficacious therapies are not available for the cure of both gliomas and glioneuronal tumors, which represent the most numerous and heterogeneous primary cancers of the central nervous system (CNS), and for neoplasms of the peripheral nervous system (PNS), which can be divided into benign tumors, mainly represented by schwannomas and neurofibromas, and malignant tumors of the peripheral nerve sheath (MPNST). Increased cellular oxidative stress and other metabolic aspects have been reported as potential etiologies in the nervous system tumors. Thus polyphenols have been tested as effective natural compounds likely useful for the prevention and therapy of this group of neoplasms, because of their antioxidant and anti-inflammatory activity. However, polyphenols show poor intestinal absorption due to individual intestinal microbiota content, poor bioavailability, and difficulty in passing the blood–brain barrier (BBB). Recently, polymeric nanoparticle-based polyphenol delivery improved their gastrointestinal absorption, their bioavailability, and entry into defined target organs. Herein, we summarize recent findings about the primary polyphenols employed for nervous system tumor prevention and treatment. We describe the limitations of their application in clinical practice and the new strategies aimed at enhancing their bioavailability and targeted delivery.

## 1. Introduction

Primary tumors of the central nervous system (CNS) are a complex heterogeneous group of benign and malignant cancers, each with their unique biology, prognosis, and sensitivity to the proposed therapies. As a general rule, brain tumors are named according to the non-neoplastic cell types that they most closely resemble and/or to their location where they are located in the brain, as classified by the World Health Organization (WHO) [1]. 

Gliomas and glioneuronal tumors are the most frequent *heterogeneous group of* primary tumors of the CNS. Gliomas, representing about 30% of the whole CNS cancers and 80% of malignant brain tumors [2], develop from glial cells, so called because the ancients thought that these cells served as “glue” between neurons (glia = glue in Greek). It is actually a group of tumors, including astrocytomas, oligodendrogliomas, ependymomas, and mixed gliomas. *Gliomas* can be aggressive (high degree of malignancy) or have a more indolent behavior (low degree of malignancy). The highest-grade astrocytomas are known as glioblastoma. Non-glial tumors constitute the bulk of neoplasms encountered in the CNS. They include a wide variety of tumor types and a spectrum of behavior ranging from indolent *benign* to highly invasive. 

Tumors of the peripheral nervous system (PNS) *can be divided into* benign *tumors, mainly represented by* schwannomas and neurofibromas, and malignant tumors of the peripheral nerve sheath (MPNST), which are a type of sarcoma with very low-frequency. Arising from the soft tissue that surrounds nerves, they develop sporadically or in a particular genetic context. Indeed, neurofibromas are part of the diagnostic criteria inclusion for neurofibromatosis type 1 (NF1), also named von Recklinghausen disease (Table 1) [3,4].

Similarly, the MPNST develop in 50% of cases in a context of NF1. On the other hand, schwannomas, especially tumors of acoustics, are a major diagnostic criterion for neurofibromatosis type 2 (NF2) (Table 2) [5].

A recently published survey of the global incidence of brain cancer estimated that between 1990 and 2016 its worldwide incidence increased most in populations grouped in the low quintile of SDI, a socio demographic index indicator of income per capita, educational level, and total fertility rate. Moreover, it is proposed that significant differences in the incidence of CNS cancer between various geographical regions can be due not only to variations in diagnoses and reporting practices but also to genetic and environmental risk factors not yet identified [6].

Unfortunately, the sole accurate targeting of genetic lesions has been shown to be an incomplete strategy, unable to extend the survival of brain tumor patient [7,8]. In the past time, cancer has been considered as a set of diseases that are caused by the accumulation of genetic mutations, and that aberrant regulation of epigenetic mechanisms may lead to human diseases, including cancer. Contrary to genetic mutations, epigenetic modifications are reversible. For this reason, epigenetic alterations are considered more effective therapeutic targets. Indeed, recent studies confirm the relevance of diet and bioactive dietary compounds for the prevention of epigenetic alterations in cancer. In fact, while former epidemiological studies did not support any or very little association between consumption of vegetable and fruit and reduced risk of cancer, clinical studies based on case-control analysis as well as data produced by large clinical cohort studies indicate an inverse correlation between the incidence of certain types of cancer and the regular consumption of fruits and vegetables [9]. For this reason, it has been hypothesized that only certain types of fruits and vegetables, likely those containing polyphenols, can exert a protective effect against cancer [9]. Correspondingly, several studies investigating the effect of diet on cancer risk and cancer progression provided evidence that polyphenols derived from tea, red wine, cocoa, certain fruits, and olive oil have an impact on carcinogenesis and tumor progression [10]. Several polyphenols, in fact, exhibit potent anti-tumor activity through their capability to reverse epigenetic alterations leading to oncogene activation and down-regulation of tumor suppressor genes [11] by interacting with oxidative reactive intermediates [12] and mutagens [13], modulating the signaling molecules involved in cell-cycle regulation [14], regulating the expression of cancer-related genes [15], or inducing apoptosis [16,17].

Furthermore, the transition from normal to cancer cell is characterized by altered cellular energy metabolism [18,19]. Indeed, the energy metabolism drives the cascade of events which lead a cell to proliferate or to die.

It is known, for example, that tumors are characterized by enhanced glucose uptake and an increased glycolysis rate [9]. Indeed, polyphenols act as inhibitors of glucose absorption and metabolism in cancer [20,21].

Oxidative stress is a common alteration present in adult-onset brain tumors as well as in hereditary cancer of the nervous system, such as Neurofibromatosis type 1 (NF1) and Tuberous Sclerosis (TSC) [22,23]. Since polyphenols show a powerful antioxidant activity, several studies have analyzed their therapeutic potential to counteract tumor progression [23].

Taking into account recent studies, the present review aims to provide up-to-date data on the effect of polyphenols in preventing the progression of central and peripheral nervous system tumors, which helps to explore their therapeutic values for future clinical settings.

## 2. Polyphenols

**Figure 1:**
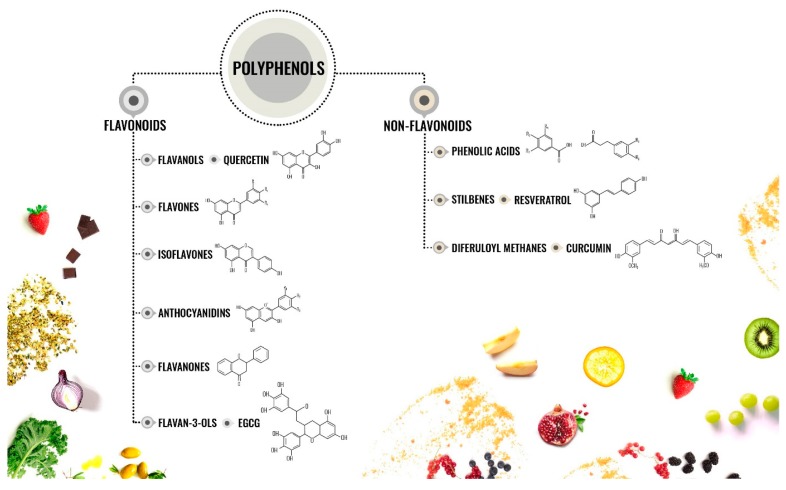
Chemical structures of different subtypes of polyphenols.

Phenols are “molecules possessing at minimum one aromatic ring with one or more hydroxyl groups attached” [23]. They are subdivided into flavonoids and non-flavonoids [24] (Figure 1). 

Flavonoids. They contain 15 carbons and two aromatic rings linked by a three-carbon bridge. This class includes: flavanols, flavones, isoflavones, flavanones, anthocyanidins, and flavan-3-ols [24].

- Flavanols are represented in the whole plant kingdom excluding the algae. Flavonols include kaempferol, quercetin, isorhamnetin, and myricetin [24].
- Flavones are contained in some herbs, such as Celery (*Apium graveolens*) and parsley (*Petrosilinum hortense*). They include apigenin, luteolin, wogonin, and baicalein [24].
- Isoflavones are contained in leguminous plants. Soybeans (*Glycine max*) present a large amount of daidzein and genistein [24]. Chickpeas are enriched in biochanin A, while peanuts contain high levels of genistein. Since isoflavones show a structure similar to estrogens, they are also defined as phytoestrogens [24].
- Flavanones are contained at elevated levels in the flavedo of citrus fruits. Hesperetin-7-O-rutinoside is also called hesperidin and is the most common member of this subclass and it is present also in apricots, plums, and bilberries [24].
- Anthocyanidins are plant pigments, which react with organic acids and sugars, leading to the formation of molecules of different colors. The major components of this subclass are peonidin, pelargonidin, petunidin, cyanidin, delphinidin, and malvidin [24].
- Flavan-3-ols. They can range from monomers (epicatechin and catechin) to oligomeric and polymeric proanthocyanidins (also called tannins). High concentrations of epicatechin-3-gallate (ECG) and epigallocatechin-3-gallate (EGCG) are present in green tea (*Camelia sinensis*) [24].

Non-flavonoids. They include the C_6_-C_1_ phenolic acids, which have a dietary relevance. The most common is the gallic acid, which is contained in numerous fruits and plants. Ellagitannins are contained in strawberries (*Fragaria ananassa*), raspberries (*Rubus idaeus*), blackberries (*Rubus spp*), persimmon (*Diospyros kaki*), pomegranates (*Punica granatum*), hazelnuts (*Corylus avellana*), and walnuts (*Juglans regia*) [24]. Belonging to this class are also: (i) secoiridoids and ligstroside, which are present in olive oil, (ii) stilbenes, whose major representing is resveratrol and is found in wine, and (iii) diferuloylmethanes that include curcumin, a curcuminoid of turmeric (Curcuma longa) [23].

## 3. Mechanisms of Cancer Modulation by Polyphenols

### 3.1. Regulation of Glucose Homeostasis

High proliferating tumor cells need to produce extra ATP to maintain their energy status, produce an increased biosynthesis of macromolecules, and maintain the cellular redox status [25]. For this reason, tumor cells need to reprogram the own metabolic flux with the aim of obtaining a surplus of ATP that is needed for an increased rate of proliferation [25]. Indeed, cancer cells are characterized by metabolic reprogramming involving an altered energy metabolism, called the Warburg effect, that shows elevated uptake and consumption of glucose and also enhanced creation of lactate buildup despite the presence of oxygen [26]. This metabolic change in tumor cells promotes cell proliferation and tumor progression by generating an increased level of glycolytic intermediates needed for the synthesis of new molecules. In addition, it augments the metabolism of glucose, which counteracts the excess metabolic formation of reactive oxygen species (ROS) in cancer cells [27]. Because of the necessity of extra ATP, glucose deprivation displays a higher cytotoxic effect on several cancer cells compared to normal cells. For this reason, inhibitors of glucose uptake and of oxidative metabolism (glycolysis inhibitors) are considered as therapeutic strategies against cancer [26]. Importantly, the Warburg effect, also defined as “aerobic lactatogenesis” [28], is considered an excellent target against cancer because therapeutic strategies directed against this metabolic shift will induce less negative side effects, leading to the reduction of treatment-associated morbidities. Indeed, several drugs targeting glucose metabolism are in trial or already approved compounds to treat cancer [29].

Several polyphenols exert an inhibitory effect on various steps of energy pathways in cancer cells, including inhibition of the uptake of glucose and block of enzymes involved in glucose metabolism. Polyphenols interfere with the glucose transporters in various cell types, affecting glucose uptake. Gossypol decreases glucose uptake in various cell types by a competitive mechanism [30]. Naringenin inhibits basal as well insulin-induced uptake of glucose in tumoral cells by blocking PIP3/Akt and MAPK activity [31]. This inhibition of glucose uptake results in an anti-proliferative effect of naringenin treatment in cancer cells [31]. Genistein reduces glucose uptake in cancer cell lines [32]. Resveratrol inhibits glycolysis in cancer cells, where it also blocks glucose uptake by lowering the expression of the glucose transporter GLUT1 [33]. Resveratrol reduces cell viability in cancer cells. Such resveratrol-dependent reduction of GLUT1 depends on a reduction of ROS and subsequent down-regulation of the transcription factor HIF-1 -alpha [33]. Moreover, resveratrol reduces cell viability by directly inhibiting the 6-Phosphofructo-1-kinase-1 (PKF), an essential enzyme of the glycolytic pathway, leading to a decreased glucose utilization and reduced ATP levels in cancer cells [34]. The flavone hesperetin reduces basal glucose uptake in cancer cells by down-regulating GLUT1 [35]. It also impairs insulin-dependent glucose uptake in cancer cells by impairing GLUT4 translocation to the plasma membrane [35]. The flavonoids epigallocatechin-3-gallate (EGCG) and quercetin reduce glucose uptake and lactate production in tumoral cells [36]. The reduction of glucose uptake is independent from signaling pathways modulated by PKC, PKG, PKA, and calcium-calmodulin [36]. The reduction of glucose uptake and lactate production due to these flavonoids results in a cytotoxic and anti-proliferative effect [36]. Luteolin reduces the glycolytic flux in cancer cells but does not affect the glucose uptake [37]. 1,2,3,4,6-penta-O-galloyl-beta-d-glucose (PGG) down-regulates the genes encoding enzymes of the pyruvate metabolism, such as acylphosphatase, pyruvate carboxylase, and aldehyde dehydrogenase (ALDH3B1) in cancer cells [38]. 

### 3.2. Modulation of Advanced Glycation Endproducts (AGEs) and their Receptor (RAGE)

Aging, altered metabolism, and diet induce the formation of advanced glycation endproducts (AGEs), which participate in the progression of several diseases, including cancer [39,40,41,42,43]. Additionally, members of the AGEs family, such as N-carboxymethyllysine (CML) and argpyrimidine, are elevated in several tumors and are implicated in cancer progression [43]. Several polyphenols inhibit AGEs formation both in vitro and in vivo. Indeed, Tomato paste blocks the formation of AGEs [44], tea polyphenols interfere with AGEs formation in physiological conditions [45], and EGCG inhibits AGEs formation [46]. The receptor for AGEs (RAGE) is also involved in cancer cell invasion and metastasis [47]. Polyphenols interfere with RAGE activation and signaling. EGCG inhibits RAGE activation [48]. Quercetin reduces RAGE expression in cancer cells, inducing cell cycle arrest, autophagy, apoptosis, and chemo-sensitivity [49].

### 3.3. Modulation of Oxidative Stress and Related Signaling Pathways

Reactive oxygen species (ROS) induce tumor formation by producing genetic mutation, inducing oncogenes, and promoting oxidative stress. The last has an effect on cell proliferation, apoptosis, and survival. Tumor cells are characterized by enhanced ROS production, leading to redox unbalance. ROS induce the oxidation of protein cysteine residues, which may induce cellular proliferation [50]. ROS play a key function in tumor initiation, promotion, and progression [51]. ROS activate oncogenes, such as Ras and c-Myc, and promote p53-dependent DNA repair [52]. In addition, ROS promote cancer progression also by inducing the activation of several signaling pathways, such as the phosphoinositide-3-kinase (PI3K)/protein kinase B (AKT), mitogen-activated protein kinase (MAPK)/extracellular-signal-regulated kinase (ERK), the inhibitor of kappa B (IκB), kinase (IKK)/nuclear factor κB (NFκB), the protein kinase D (PKD), JNK, and PI3K, which in turn modulate the activity of several transcription factors that participate to cancer initiation/progression [52]. 

Polyphenols possess both anti-oxidant and pro-oxidant activity, which modulate cell proliferation and apoptotic pathways [53]. Quercetin scavenges superoxide anions, leading to the formation of H_2_O_2_, semiquinone, and quercetin radicals. The latter deplete the intracellular anti-oxidant systems [52]. Indeed, quercetin leads to ROS-dependent apoptosis, necrosis, and autophagy in cancer cells [52]. In addition, quercetin induces cell cycle arrest by modulating p21^WAF^, cyclin B, and p27^KIP1^ in cancer cells [52]. Notably, curcumin exerts opposite effects in cancer cells compared to its effects in normal cells, suggesting that curcumin can be beneficial in preventing cancer without affecting the homeostasis of normal cells. In normal cells, curcumin acts as an anti-oxidant by scavenging hydroxyl radicals, superoxide, nitric oxide, H2O2, and peroxynitrite [54]. In addition, curcumin regulates the expression of HO-1, GPX, and SOD in normal cells [54]. On the other hand, in cancer cells curcumin has a pro-oxidative effect that induces apoptosis [55]. Indeed, curcumin induces ROS formation in cancer cells [56,57]. Curcumin’s pro-oxidative activity occurs in the mitochondrial membranes of cancer cells, leading to depolarization of mitochondrial and down-regulation of ATP synthesis, ultimately inducing apoptosis [58]. Curcumin-dependent ROS production in cancer cells induces Erk1/2 and p38 MAPK pathways that lead to autophagy [59]. Curcumin blocks the survival and proliferation of cancer stem cell through a ROS-mediated inhibition of NFκB and STAT3 in glioblastoma [60]. Capsaicin has been shown to exert beneficial effects against glioma. Indeed, in glioma, capsaicin (trans-8-methyl-N-vanillyl-6-nonenamide) affects the mitochondrial membrane potential, leading to ROS elevation and subsequent caspase 3 activation [61]. Capsaicin not only induces apoptosis but also cell cycle arrest [62]. EGCG possesses both anti-oxidant and pro-oxidant activity. It produces ROS by auto-oxidation [63]. EGCG induces apoptosis by inhibiting the PI3k/AKT pathway. In addition, it decreases the mitochondrial membrane potential, leading to apoptosis [52]. Phenethyl isothiocyanate (PEITC) decreases intracellular GSH, leading to enhanced ROS accumulation and mitochondrial dysfunction exclusively in cancer cells [64]. Benzyl isothiocyanate (BITC) induces oxidative stress in glioma cells by exhausting SOD and GSH levels, leading to caspase-mediated apoptosis [65]. Piperine blocks tumor growth by inducing oxidative stress, mitochondria dysfunction, and subsequent apoptosis [66]. Resveratrol inhibits tum initiation and progression by inducing apoptosis in neuroblastoma cells [67]. It promotes apoptosis by inducing the death receptors for TRAIL and FasL, ROS-dependent caspase activation, and p53 [68]. P-Coumaric acid (p-CoA) promotes apoptosis in cancer cells by enhancing ROS formation and inducing mitochondrial depolarization [69]. Naringenin promotes apoptosis in cancer cells through induction of ROS formation, which in turn leads to p38 MAPK-dependent caspase activation [70]. Gallic acid inhibits cancer cell growth by inducing ROS production [71]. Thus, we may conclude that several polyphenols counteract tumor progression by inducing cancer-specific ROS production.

### 3.4. Other Mechanisms

Polyphenols modify the metabolism of pro-carcinogens through mechanisms that alter the levels of cytochromes P450 (CYPs), which plays an essential role in cancer promotion [72]. Polyphenols produce epigenetic changes that have a preventive effect against cancer [73]. Polyphenols also show a preventive effect by inhibiting inflammation [74]. They also modulate the autophagy flux [75].

Polyphenols prevent metastasis formation by affecting the activity of urokinase and matrix metalloproteinases and by inhibiting angiogenesis through modulation of the vascular endothelial growth factor expression and receptor phosphorylation [76].

In addition, dietary polyphenols are employed together with conventional pharmacological therapy or cytotoxic agents used to treat drug-resistant cancer cells [74].

Several studies indicated herein tested the efficacy of polyphenols at concentrations equivalent to oral sub ministration of juices/extracts of natural substances containing polyphenols (range 100–800 mg/day), while other studies analyzed higher concentrations (range 10–75 μM). Notably, concentrations of polyphenols equivalent to diet intake show also beneficial effects.

## 4. Polyphenol Effects on Central Nervous System Cancers 

Several risk factors are involved in the onset and progression of adult brain tumors: aging, diet, environmental exposure, head trauma, infections, and cigarette smoking [77]. Primary brain tumors are named gliomas and classified according to their putative original cell type. Glioblastoma multiform (GBM) shows the highest aggressive phenotype (grade IV) representing the 60% of age-related brain tumors [78]. At present there is not definite cure for GBM; thus, researchers are looking at innovative therapeutic strategies [23].

Recent studies in vitro and in vivo have underlined the therapeutic efficacy against cancer of several polyphenols, such as quercetin, epigallocatechin-3-gallate (EGCG), resveratrol, and curcumin [79]. Moreover, it has been reported that the polyphenols’ therapeutic potential is further enhanced when they are used in combination or added to pharmaceutical compounds [74,79].

### 4.1. Curcumin Effects on Central Nervous System Cancers 

The effective anti-tumor activity of curcumin treatment in GBM has been shown by several studies [23,80]. Curcumin exerts a pro-differentiative effect in glioma-stem cells because of its activation of the autophagy flux [81]. In human glioblastoma T98G cells, this polyphenol induces the activation of both receptor-mediated and mitochondria-mediated proteolytic pathways, which in turn promote apoptosis [55]. Curcumin down-regulates the expression of cancer signaling pathways (i.e., Notch1 and pAKT), leading to blockade of cell growth, apoptosis, and inhibition of migration and invasion [82]. In human glioma cells, it lowers the protein levels of neuronal precursor cell-expressed developmentally down-regulated 4–1 (NEDD4) [82]. Delivery of curcumin into the brain of GBM mice produces the remission of tumor in 50% of the animals and modifies the phenotype of the microglia surrounding the tumor [83]. Curcumin exerts an efficient induction of autophagy [75], which can lead to apoptosis in cancer cells. Moreover, curcumin treatment in A172 human glioblastoma cells leads to cell death by inducing autophagy flux [84]. Another study confirmed that curcumin promotes autophagy in glioblastoma cells while it inhibits mitophagy [85]. Curcumin also blocks invasion and migration potential of glioblastoma U87 cells by decreasing the expression of fascin, an actin-binding protein involved in migration and invasion [86]. Noteworthy, curcumin exerts a radiosensitizing effect on GBM [87]. Moreover, curcumin acts as a photosensitizer in sNB-19 glioblastoma cells, showing that it can be used to improve the photodynamic therapy for GBM treatment [88].

### 4.2. Resveratrol Effects on Central Nervous System Cancers 

Several studies demonstrate the efficacy of resveratrol in lowering tumorigenesis and preventing metastasis [23,89,90]. Resveratrol has a powerful capability of down-regulating the self-renewal and tumor-initiating capability of glioma stem cells obtained from GBM patients by inducing the p53/ p21 pathway [91]. Resveratrol possesses a potent effect in inhibiting the invasion and migration capability of glioblastoma cells by activating the RhoA/ROCK pathway [92]. Resveratrol decreases cell growth and motility, enhances cell death, and interferes with the epithelial-mesenchymal transition modulating the Wnt signaling pathway [93]. Resveratrol lowers tumorigenic potential and improves the effects of radiotherapy in vitro and in vivo against GBM-derived tumor stem cells to by inhibiting the signal transducer and activator of transcription 3 (STAT3) [94]. Resveratrol blocks the growth of U-87MG glioblastoma cells and lowers the expression of human telomerase reverse transcriptase (hTERT) as well as the catalytic subunit of the telomerase and a biomarker of cell immortalization, confirming that resveratrol can be used as a therapeutic agent for GBM [95]. The postoperative administration of resveratrol results in a significant prognosis amelioration of rat-advanced orthotopic glioblastoma by reducing growth, inducting apoptosis, and suppressing STAT3 signaling [96]. In addition, resveratrol blocks epithelial-mesenchymal transition in GBM by modulating Smad signaling [97].

In combination with Paclitaxel, resveratrol enhances the oxidant and apoptotic effect of the pharmacological compound by activating the TRPM2 channel in glioblastoma cells [98].

### 4.3. EGCG Effects on Central Nervous System Cancers 

Several studies show the beneficial effect of EGCG as a therapeutic agent for brain tumors [23]. EGCG exerts an inhibitory effect in three glioma cell lines by modulating the epidermal growth factor-1 (EGF-1) [99]. EGCG potentiates the effects of ionizing radiation (IR) in GBM by modulating the activity of Ras homolog gene family member A (RhoA) and survivin, with the last being involved in the regulation of apoptosis. Treatment with EGCG combined with radiotherapy ameliorates the efficacy of IR treatments [100]. EGCG also enhances the anti-cancer activity of cytotoxic agents [101]. Indeed, in a mouse model of glioblastoma, EGCG enhances the anti-cancer potential of temozolomide, which promotes DNA damage [101]. Treatment with EGCG alone or in combination with temozolomide affects glioma stem cell survival and migration capability, as well as inhibits neurosphere formation [102]. In addition, such treatments induce apoptosis by down-regulating p-Akt and Bcl-2 [102]. In human glioblastoma U251 cells, EGCG promotes apoptosis and blocks cell-growth because of inhibiting the JAK2/STAT3 signaling pathway [103]. EGCG suppresses the invasion properties of human glioblastoma T-98G cells by down-regulating MMP-2 and MMP-9 expression [104]. Interestingly, EGCG inhibits the effects of the glucose-regulated protein 78 (GRP78), which is up-regulated in GBM by direct protein–protein interaction that results in a conformational change in GRP78, probably leading to its inactivation [105]. At the low concentration of 100 nM, EGCG activates endogenous repair pathways while at higher concentrations, EGCG induces ROS production and autophagy [106]. These data suggest that drinking green tea containing low concentrations of EGCG may exert a chemo-preventive effect against GBM, while higher concentrations (500 μM) show a therapeutic effect [106]. Importantly, EGCG inhibits the expression of O^6^-Methylguanine DNA-Methyltransferase (MGMT) in GBM-derived cells only, which is an essential regulator of the resistance to temozolomide (TMZ) in glioblastomas. EGCG treatment in two GBM cell lines (GBM-XD and T98G) results in suppression of MGMT expression, abolishes TMZ resistance, and prevents β catenin translocation into the nucleus [107]. On the contrary, the addition of EGCG to non-tumor glial cell culture (GliaX) enhances MGMT expression by inhibiting the methylation of the MGMT promoter [107]. Recently, it has been shown that EGCG induces telomere shortening in U251 glioblastoma, leading to senescence [108]. In addition, it also promotes telomere-independent genotoxicity [108].

### 4.4. Quercetin Effects on Central Nervous System Cancers 

The therapeutic potential of quercetin for the cure of GBM has been extensively analyzed [23]. The glycoside form of quercetin called Rutin exerts an anti-proliferative effect on human GBM cells [109]. Rutin reduces the survival and proliferation of GL-15 cell lines, resulting in a decrement of phosphorylated extracellular signal-regulated protein kinases 1 and 2 (ERK1/2), which exert an essential role in cell proliferation and apoptosis modulation [109]. In GBM cell cultures, rutin induces astroglial differentiation and apoptosis [109]. In GBM cultures, treatment with quercetin alone or together with temozolomide, induces apoptosis, whereas it does not affect autophagy [110]. Treatment with quercetin or its addition during with irradiation promotes apoptosis. This anti-cancer effect is due to activation of caspase-3 and poly [ADP-ribose] polymerase 1 (PARP-1), which are concomitant to the inhibition of the Akt pathway [111]. In GBM cells, co-treatment with rutin and temozolomide results in enhanced cytotoxicity because of inhibition of the autophagy flux [112]. Studies in subcutaneous and orthotopic xenograft using concomitant treatment with temozolomide and rutin show a decreased tumor volumes, while treatment with temozolomide or rutin alone is less effective [112]. In U251 glioblastoma human cells, quercetin inhibits cell proliferation and viability as well as invasion and migration properties [113,114]. Quercetin shows a pro-apoptotic effect also because it regulates the expression of apoptotic genes and because it induces the cell cycle arrest [113]. Using U87MG, C6, and U138 glioblastoma cultures, we demonstrated that the water extract of *Ruta graveolens* L. promotes cell death. We also found that rue activates ERK1/2 and AKT, resulting in an inhibition of cell growth. We also show that rutin, the major component of the *Ruta graveolens* water extract, is unable to induce cell death [115]. Quercetin in combination with sodium butyrate promotes apoptosis in rat C6 and human T98G GBM cells by inhibiting autophagy [116].

## 5. Polyphenol Effects on Tumors of the Peripheral Nervous System 

Neurofibromatosis type 1 (NF1) is an autosomal dominant disorder showing complex phenotypes and it is caused by inherited mutations in the NF1 gene, which is a tumor suppressor. Almost all NF1 patients develop pigmentary lesions (café-au-lait macules, skinfold freckling, and Lisch nodules) and dermal neurofibromas (Table 1). In some patients are also present brain tumors (glioblastoma and optic pathway gliomas), peripheral nerve tumors (plexiform neurofibromas, spinal neurofibromas, and malignant peripheral nerve sheath tumors), skeletal abnormalities (tibial pseudarthrosis, orbital dysplasia, and scoliosis), attention deficits, learning disabilities, and social and behavioral problems, which impair the quality of life [117].

Neurofibromatosis type 2 (NF2) is a genetic disorder characterized by the presence of multiple benign tumors of the peripheral and central nervous system (including meningiomas, schwannomas, and ependymomas) (Table 2). 

NF2 patients are almost always diagnosed late in life, around the second or third decade of life [118,119]. NF2 is characterized by the presence of benign tumors. However, such tumors can induce mortality that is associated to the location of the tumors as well as can be promoted by the treatments. Currently, the only therapy available consists in a local treatment of the tumors and is not effective. Thus, there is a need to develop systemic therapies aimed to improve the outcome of NF2 [23,119].

It is well documented that a healthy diet including a high consumption of fruit and vegetables has a preventive effect against cancer and results in a lower incidence of tumor development and tumor-induced mortality [120].

Recently, researchers started to investigate the chemopreventive and/or chemotherapeutic potential of polyphenolic compounds [120]. Since polyphenols possess anti-oxidant proprieties, their consumption has a beneficial effect against the high levels of oxidative stress produced by cancer cells [51]. 

### 5.1. Curcumin Effects on Tumors of the Peripheral Nervous System

Curcumin reduces proliferation and enhances the apoptosis rate in HEI-193 human schwannoma cells [118]. These results indicate that administration of curcumin to patients with NF2 schwannomas may exert a beneficial effect. We describe the first experience with curcumin supplementation in NF1 patients. We show that a therapeutic strategy involving a high adherence to the Mediterranean diet together with the administration of 1200 mg/day of curcumin results in a significant reduction of the number and volume of cutaneous neurofibromas in NF1 patients [121]. Notably, we demonstrate by Magnetic Resonance Imaging that in one patient this therapeutic strategy results in a sensible reduction in volume (28%) of a large cranial plexiform neurofibroma. On the contrary, administration of curcumin in association with a Western diet has not effect on NF1 tumors, suggesting that some components in the Mediterranean diet may improve curcumin bioavailability and activity [121]. A recent study revealed that calebin-A, derived from turmeric *Curcuma longa*, (a) inhibits the cell growth in the malignant peripheral nerve sheath tumor (MPNST) transformed from NF1-related plexiform neurofibroma, and (b) blocks cell growth in primary neurofibroma cells [122]. Calebin-A induces the cell cycle arrest and decreases hTERT, phosphorylated- ERK1/2, -AKT, and surviving [122].

### 5.2. EGCG Effects on Tumors of the Peripheral Nervous System

Only one study reported that EGCG reduces the proliferation of an MPNST transformed from NF1-related plexiform neurofibroma [122].

## 6. Bioavailability of Dietary Polyphenols

Several studies have indicated that dietary polyphenols exert neuroprotective functions. However, their clinical application is still limited. In fact, polyphenols exert poor effect in vivo when compared to their activity in vitro [123,124]. The difference between in vitro and in vivo effects of polyphenols is mainly associated to their poor absorption, rapid metabolism, and massive system elimination, which represent a limitation regarding their therapeutic action and clinical application [125]. Several studies underline that the chemical structure of polyphenols plays a key role in modulating the rate and extent of their absorption upon ingestion [74]. Noteworthy, the individual variability in drug absorption and metabolism has a key role in modulating the effects of polyphenols in vivo. Indeed, the absorption is regulated by the local microflora, by the metabolic activity and by the hepatic function [126]. In addition, the CNS is protected by the blood brain barrier (BBB), which regulates the transport of molecules into the CNS. Thus, the transport across the BBB further limits the therapeutic potential of dietary polyphenols. For this reason, several ongoing research projects are studying how to improve the bioavailability of polyphenols [125]. These studies aim at ameliorating the biochemical stability and transport across the BBB of the polyphenols as well as decreasing their degradation [125]. Another study investigated the transport across the BBB of bioavailable phenolic sulfates derived from the colonic metabolism of berries [127]. They found that these compounds show a differential transport across the BBB, which was related to their chemical structure. In addition, they discovered that these compounds were further metabolized by the endothelial cells, leading to the production of novel molecules with potential bioactivity [127]. This study also demonstrated that pre-treatment with these compounds (a) ameliorated the response to oxidative stress and toxicity and (b) reduced the inflammatory response by modulating NF-kB activity [127]. Thus, this study demonstrated that these polyphenols cross the BBB and exert a neuroprotective and anti-inflammatory function. Furthermore, it has been shown that the gut microbiota metabolize the dietary polyphenols, promoting the production of bioactive molecules that cross the BBB and modulate the neuronal function by acting as neurotransmitters [128]. Interestingly, dietary polyphenols modulate the bacterial composition of the gut microbiota, acting on the microbiota-gut-brain axis, which is considered as a neuroendocrine system [128]. These studies support the hypothesis that dietary polyphenols exert a beneficial effect by modulating the gut microbiota, leading to a neuroprotective effect via the gut-brain axis. Thus, they may have a therapeutic role in the prevention of diseases affecting the nervous system [128].

However, several challenges remain. These include (a) the exploration of the therapeutic interplay between polyphenols or other natural substances contained in the Mediterranean diet [121,129], (b) their molecular characterization, and (c) the definition of optimal absorption levels and bioavailability improvement. This is necessary to ensure therapeutic efficacy, that these substances cross the intestinal and blood–brain barriers, and that matrices can be developed for the release of product formulations. Indeed, nanotechnology can provide new materials for the delivery of polyphenols, improving their absorption and efficacy [130]. These technologies can provide food-based nanodelivery vehicles with different surface properties. To date, several nanovehicles, such as nanoemulsions, protein-polysaccharide coacervas, liposomes, and small cochlear structures, are produced only on a laboratory scale. In the future these systems will have applications in the development of functional foods at an industry scale [131].

## 7. Clinical Trials

To date, the US National Institute of Health database shows only two completed clinical trials using curcumin and polyphenols for the treatment of GBM (http://www.clinicaltrial.gov/; searching for: “Glioblastoma multiforme” and “Curcumin” and “polyphenols”; and http://www.clinicaltrial.gov/; searching for: “Glioblastoma multiforme” and “Curcumin”). There are no any curcumin-based clinical trials for NF2 or NF1 treatments (http://www.clinicaltrial.gov/; searching for: “Neurofibromatosis type 2” and “Curcumin”).

## 8. Conclusions

The incidence of brain tumors has been increasing recently. Despite considerable efforts to find an effective therapy, the treatment of some cancers of the nervous system still remains a challenge in a war in which, thus far, few battles have been won. The numerous metabolic aspects underlying the tumors of the CNS and PNS have opened the way to new therapeutic approaches that see an interesting therapeutic strategy in diet and, in particular, foods with anti-oxidant activity. In particular, several studies have underlined the beneficial effect of dietary polyphenols for the prevention of tumors of the CNS and PNS. 

Furthermore, recent studies have revealed the positive effect of polyphenols on the microbioma-intestine-brain axis, demonstrating the therapeutic potential of dietary polyphenols in the prevention of diseases affecting the nervous system.

However, the low bioavailability of dietary polyphenols is still a limitation for their introduction into clinical practice. A promising solution lies in polymeric nanoparticle-based polyphenol delivery systems that prevent the degradation of bioactive compounds and enhance their absorption and bioavailability. 

## Figures and Tables

**Table 1:** Criteria for the clinical diagnosis of NF1 *(At least two are required).*

Criterion	Features
Six or more café-au-lait macules	>5 mm before puberty >15 mm after puberty
Freckling	Axillary, inguinal
Neurofibromas	Two or more neurofibromas or one plexiform neurofibroma
Skeletal dysplasia	Sphenoid or tibial lesion
Lisch nodules	Two or more iris hamartomas
Optic glioma	Detected by neuroimaging (usually MRI)
First degree relative with NF1	Sibling or parent with NF1
Skeletal dysplasia	Sphenoid or tibial lesion

**Table 2:** Diagnostic criteria for Neurofibromatosis type 2 (these include the National Institutes of Health (NIH) criteria with additional criteria).

Main Criteria	Additional Criteria
Bilateral vestibular schwannomas (VS) or family history of NF2 *plus* (1) Unilateral VS *or* (2) Any two of: meningioma, glioma, neurofibroma, schwannoma, posterior subcapsular lenticular opacities	Unilateral VS *plus* any two of: meningioma, glioma, neurofibroma, schwannoma, and posterior subcapsular opacities or Multiple meningioma (two or more) plus unilateral VS or any two of: glioma, neurofibroma, schwannoma, and cataract
NF2—Neurofibromatosis type 2; VS—vestibular schwannomas	

## References

[1] Louis D.N., Perry A., Reifenberger G., von Deimling A., Figarella-Branger D., Cavenee W.K., Ohgaki H., Wiestler O.D., Kleihues P., Ellison D.W. (2016). The 2016 World Health Organization Classification of Tumors of the Central Nervous System: A summary. Acta Neuropathol..

[2] Goodenberger M.L., Jenkins R.B. (2012). Genetics of adult glioma. Cancer Genet..

[3] National Institutes of Health (1988). Consensus Development Conference Statement: Neurofibromatosis. Bethesda, Md., USA, July 13–15, 1987. Neurofibromatosis.

[4] Giugliano T., Santoro C., Torella A., Del Vecchio Blanco F., Grandone A., Onore M.E., Melone M.A.B., Straccia G., Melis D., Piccolo V. (2019). Clinical and Genetic Findings in Children with Neurofibromatosis Type 1, Legius Syndrome, and Other Related Neurocutaneous Disorders. Genes.

[5] Evans D.G. (2009). Neurofibromatosis type 2 (NF2): A clinical and molecular review. Orphanet. J. Rare Dis..

[6] GBD 2016 Brain and Other CNS Cancer Collaborators (2019). Global, regional, and national burden of brain and other CNS cancer, 1990–2016: A systematic analysis for the Global Burden of Disease Study 2016. Lancet Neurol..

[7] Dhez A.C., Benedetti E., Antonosante A., Panella G., Ranieri B., Florio T.M., Cristiano L., Angelucci F., Giansanti F., Di Leandro L. (2018). Targeted therapy of human glioblastoma via delivery of a toxin through a peptide directed to cell surface nucleolin. J. Cell Physiol..

[8] Mack S.C., Hubert C.G., Miller T.E., Taylor M.D., Rich J.N. (2016). An epigenetic gateway to brain tumor cell identity. Nat. Neurosci..

[9] Vauzour D., Rodriguez-Mateos A., Corona G., Oruna-Concha M.J., Spencer J.P. (2010). Polyphenols and human health: Prevention of disease and mechanisms of action. Nutrients.

[10] Middleton E., Kandaswami C., Theoharides T.C. (2000). The effects of plant flavonoids on mammalian cells: Implications for inflammation, heart disease, and cancer. Pharm. Res..

[11] Carlos-Reyes Á., López-González J.S., Meneses-Flores M., Gallardo-Rincón D., Ruíz-García E., Marchat L.A., Astudillo-de la Vega H., Hernández de la Cruz O.N., López-Camarillo C. (2019). Dietary Compounds as Epigenetic Modulating Agents in Cancer. Front. Genet..

[12] Duthie S.J., Dobson V.L. (1999). Dietary flavonoids protect human colonocyte DNA from oxidative attack in vitro. Eur. J. Nutr..

[13] Calomme M., Pieters L., Vlietinck A., Vanden Berghe D. (1996). Inhibition of bacterial mutagenesis by Citrus flavonoids. Planta Med..

[14] Plaumann B., Fritsche M., Rimpler H., Brandner G., Hess R.D. (1996). Flavonoids activate wild-type p53. Oncogene.

[15] van Erk M.J., Roepman P., van der Lende T.R., Stierum R.H., Aarts J.M., van Bladeren P.J., van Ommen B. (2005). Integrated assessment by multiple gene expression analysis of quercetin bioactivity on anticancer-related mechanisms in colon cancer cells in vitro. Eur. J. Nutr..

[16] Fabiani R., De Bartolomeo A., Rosignoli P., Servili M., Montedoro G.F., Morozzi G. (2002). Cancer chemoprevention by hydroxytyrosol isolated from virgin olive oil through G1 cell cycle arrest and apoptosis. Eur. J. Cancer Prev..

[17] Mantena S.K., Baliga M.S., Katiyar S.K. (2006). Grape seed proanthocyanidins induce apoptosis and inhibit metastasis of highly metastatic breast carcinoma cells. Carcinogenesis.

[18] Cirillo A., Di Salle A., Petillo O., Melone M.A., Grimaldi G., Bellotti A., Torelli G., De’Santi M.S., Cantatore G., Marinelli A. (2014). High grade glioblastoma is associated with aberrant expression of ZFP57, a protein involved in gene imprinting, and of CPT1A and CPT1C that regulate fatty acid metabolism. Cancer Biol. Ther..

[19] Melone M.A.B., Valentino A., Margarucci S., Galderisi U., Giordano A., Peluso G. (2018). The carnitine system and cancer metabolic plasticity. Cell Death Dis..

[20] León D., Uribe E., Zambrano A., Salas M. (2017). Implications of Resveratrol on Glucose Uptake and Metabolism. Molecules.

[21] Siddiqui F.A., Prakasam G., Chattopadhyay S., Rehman A.U., Padder R.A., Ansari M.A., Irshad R., Mangalhara K., Bamezai R., Husain M. (2018). Curcumin decreases Warburg effect in cancer cells by down-regulating pyruvate kinase M2 via mTOR-HIF1α inhibition. Sci. Rep..

[22] Incecik F., Avcıoğlu G., Erel Ö., Neşelioğlu S., Besen S., Altunbaşak S. (2019). Dynamic thiol/disulphide homeostasis in children with neurofibromatosis type 1 and tuberous sclerosis. Acta Neurol. Belg..

[23] Squillaro T., Schettino C., Sampaolo S., Galderis U., Di Iorio G., Giordano A., Melone M.A.B. (2017). Adult-onset brain tumors and neurodegeneration: Are polyphenols protective?. J. Cell Physiol..

[24] Del Rio D., Rodriguez-Mateos A., Spencer J.P., Tognolini M., Borges G., Crozier A. (2013). Dietary (poly)phenolics in human health: Structures, bioavailability, and evidence of protective effects against chronic diseases. Antioxid. Redox Signal..

[25] Cairns R.A., Harris I.S., Mak T.W. (2011). Regulation of cancer cell metabolism. Nat. Rev. Cancer.

[26] Hanahan D., Weinberg R.A. (2011). Hallmarks of cancer: The next generation. Cell.

[27] Aykin-Burns N., Ahmad I.M., Zhu Y., Oberley L.W., Spitz D.R. (2009). Increased levels of superoxide and H2O2 mediate the differential susceptibility of cancer cells versus normal cells to glucose deprivation. Biochem. J..

[28] Martel F., Guedes M., Keating E. (2016). Effect of polyphenols on glucose and lactate transport by breast cancer cells. Breast Cancer Res. Treat..

[29] Luengo A., Gui D.Y., Vander-Heiden M.G. (2017). Targeting metabolism for cancer therapy. Cell Chem. Biol..

[30] Pérez A., Ojeda P., Valenzuela X., Ortega M., Sánchez C., Ojeda L., Castro M., Cárcamo J.G., Rauch M.C., Concha I.I. (2009). Endofacial competitive inhibition of the glucose transporter 1 activity by gossypol. Am. J. Physiol. Cell Physiol..

[31] Harmon A.W., Patel Y.M. (2004). Naringenin inhibits glucose uptake in MCF-7 breast cancer cells: A mechanism for impaired cellular proliferation. Breast Cancer Res. Treat..

[32] Lim H.A., Kim J.H., Kim J.H., Sung M.K., Kim M.K., Park J.H., Kim J.S. (2006). Genistein induces glucose-regulated protein 78 in mammary tumor cells. J. Med. Food.

[33] Jung K.H., Lee J.H., Thien-Quach C.H., Paik J.Y., Oh H., Park J.W., Lee E.J., Moon S.H., Lee K.H. (2013). Resveratrol suppresses cancer cell glucose uptake by targeting reactive oxygen species-mediated hypoxia-inducible factor-1α activation. J. Nucl. Med..

[34] Gomez L.S., Zancan P., Marcondes M.C., Ramos-Santos L., Meyer-Fernandes J.R., Sola-Penna M., Da Silva D. (2013). Resveratrol decreases breast cancer cell viability and glucose metabolism by inhibiting 6-phosphofructo-1-kinase. Biochimie.

[35] Yang Y., Wolfram J., Boom K., Fang X., Shen H., Ferrari M. (2013). Hesperetin impairs glucose uptake and inhibits proliferation of breast cancer cells. Cell Biochem. Funct..

[36] Moreira L., Araújo I., Costa T., Correia-Branco A., Faria A., Martel F., Keating E. (2013). Quercetin and epigallocatechin gallate inhibit glucose uptake and metabolism by breast cancer cells by an estrogen receptor-independent mechanism. Exp. Cell Res..

[37] Du G.J., Song Z.H., Lin H.H., Han X.F., Zhang S., Yang Y.M. (2008). Luteolin as a gly-colysis inhibitor offers superior efficacy and lesser toxicity of doxorubicin in breast cancer cells. Biochem. Biophys. Res. Commun..

[38] Yu W.S., Jeong S.J., Kim J.H., Lee H.J., Song H.S., Kim M.S., Ko E., Lee H.J., Khil J.H., Jang H.J. (2011). The genome-wide expression profile of 1,2,3,4,6-penta-O-galloyl-β-D-glucose-treated MDA-MB-231 breast cancer cells: Molecular target on cancer metabolism. Mol. Cells.

[39] Anzilotti S., Giampà C., Laurenti D., Perrone L., Bernardi G., Melone M.A., Fusco F.R. (2012). Immunohistochemical localization of receptor for advanced glycation end (RAGE) products in the R6/2 mouse model of Huntington’s disease. Brain Res. Bull..

[40] Aragno M., Mastrocola R. (2017). Dietary Sugars and Endogenous Formation of Advanced Glycation Endproducts: Emerging Mechanisms of Disease. Nutrients.

[41] Perrone L., Grant W.B. (2015). Observational and ecological studies of dietary advanced glycation end products in national diets and Alzheimer’s disease incidence and prevalence. J. Alzheimers Dis..

[42] Perrone L., Sbai O., Nawroth P.P., Bierhaus A. (2012). The Complexity of Sporadic Alzheimer’s Disease Pathogenesis: The Role of RAGE as Therapeutic Target to Promote Neuroprotection by Inhibiting Neurovascular Dysfunction. Int. J. Alzheimers Dis..

[43] van Heist J., Niessen H., Hoekman K., Schalkwij C. (2005). Advanced glycation end products in human cancer tissues: Detection of Nepsilon-(carboxymethyl)lysine and argpryrimidine. Ann. N. Y. Acad. Sci..

[44] Kiho T., Usui S., Hirano K., Aizawa K., Inakuma T. (2004). Tomato paste fraction inhibiting the formation of advance glycation end-products. Biosci. Biotechnol. Biochem..

[45] Lo C.-Y., Li S., Tan D., Pan M.-H., Sang S., Ho C.-T. (2006). Trapping reactions of reactive carbonyl species with tea polyphenols in simulated physiological conditions. Mol. Nutr. Food Res..

[46] Sang S., Shao X., Bai N., Lo C.-Y., Yang C., Ho C.-T. (2007). Tea polyphenol (−)-Epigallocatechin-3-Gallate: A new trapping agent of reactive dicarbonyl species. Chem. Res. Toxicol..

[47] Palanissami G., Paul S.F.D. (2018). RAGE and Its Ligands: Molecular Interplay Between Glycation, Inflammation, and Hallmarks of Cancer-a Review. Horm. Cancer.

[48] Takada M., Ku Y., Toyama H., Suzuki Y., Kuroda Y. (2002). Suppressive effects of tea polyphenol and conformational changes with receptor for advanced glycation en products (RAGE) expression in human hepatoma cells. Hepatogastroenterology.

[49] Lan C.Y., Chen S.Y., Kuo C.W., Lu C.C., Yen G.C. (2019). Quercetin facilitates cell death and chemosensitivity through RAGE/PI3K/AKT/mTOR axis in human pancreatic cancer cells. J. Food Drug Anal..

[50] Schieber M., Chandel N.S. (2014). ROS function in redox signaling and oxidative stress. Curr. Biol..

[51] Liou G.Y., Storz P. (2010). Reactive oxygen species in cancer. Free Radic. Res..

[52] NavaneethaKrishnan S., Rosales J.L., Lee K.Y. (2019). ROS-Mediated Cancer Cell Killing through Dietary Phytochemicals. Oxid. Med. Cell Longev..

[53] Gibellini L., Pinti M., Nasi M., De Biasi S., Roat E., Bertoncelli L., Cossarizza A. (2010). Interfering with ROS Metabolism in Cancer Cells: The Potential Role of Quercetin. Cancers.

[54] Gibellini L., Bianchini E., De Biasi S., Nasi M., Cossarizza A., Pinti M. (2015). Natural compounds modulating mitochon-drial functions. Evid. Based Complement. Altern. Med..

[55] Karmakar S., Banik N.L., Patel S.J., Ray S.K. (2006). Curcumin activated both receptor-mediated and mitochondria-mediated proteolytic pathways for apoptosis in human glioblastoma T98G cells. Neurosci. Lett..

[56] Bhaumik S., Anjum R., Rangaraj N., Pardhasaradhi B.V.V., Khar A. (1999). Curcumin mediated apoptosis in AK-5 tumor cells involves the production of reactive oxygen intermediates. FEBS Lett..

[57] Thayyullathil F., Chathoth S., Hago A., Patel M., Galadari S. (2008). Rapid reactive oxygen species (ROS) generation induced by curcumin leads to caspase-dependent and -independent apoptosis in L929 cells. Free Radic. Biol. Med..

[58] Morin D., Barthelemy S., Zini R., Labidalle S., Tillement J.P. (2001). Curcumin induces the mitochondrial permeability transition pore mediated by membrane protein thiol oxidation. FEBS Lett..

[59] Lee Y.J., Kim N.Y., Suh Y.A., Lee C. (2011). Involvement of ROS in curcumin-induced autophagic cell death. Korean J. Physiol. Pharmacol..

[60] Gersey Z.C., Rodriguez G.A., Barbarite E., Sanchez A., Walters W.M., Ohaeto K.C., Komotar R.J., Graham R.M. (2017). Curcumin decreases malignant characteristics of glioblastoma stem cells via induction of reactive oxygen species. BMC Cancer.

[61] Xie L., Xiang G.H., Tang T., Tang Y., Zhao L.Y., Liu D., Zhang Y.R., Tang J.T., Zhou S., Wu D.H. (2016). Capsaicin and dihydrocapsaicin induce apoptosis in human glioma cells via ROS and Ca2+-mediated mitochondrial pathway. Mol. Med. Rep..

[62] Lin C.H., Lu W.C., Wang C.W., Chan Y.C., Chen M.K. (2013). Capsaicin induces cell cycle arrest and apoptosis in human KB cancer cells. BMC Complement. Altern. Med..

[63] Min K.J., Kwon T.K. (2014). Anticancer effects and molecular mechanisms of epigallocatechin-3-gallate. Integr. Med. Res..

[64] Trachootham D., Zhou Y., Zhang H., Demizu Y., Chen Z., Pelicano H., Chiao P.J., Achanta G., Arlinghaus R.B., Liu J. (2006). Selective killing of oncogenically transformed cells through a ROS-mediated mechanism by beta-phenylethyl isothiocyanate. Cancer Cell.

[65] Zhu Y., Zhuang J.X., Wang Q., Zhang H.Y., Yang P. (2013). Inhibitory effect of benzyl isothiocyanate on proliferation in vitro of human glioma cells. Asian Pac. J. Cancer Prev..

[66] Rather R.A., Bhagat M. (2018). Cancer chemoprevention and piperine: Molecular mechanisms and therapeutic opportunities. Front. Cell Dev. Biol..

[67] Chen Y., Tseng S.H., Lai H.S., Chen W.J. (2004). Resveratrol-induced cellular apoptosis and cell cycle arrest in neuroblas-toma cells and antitumor effects on neuroblastoma in mice. Surgery.

[68] Shankar S., Chen Q., Siddiqui I., Sarva K., Srivastava R.K. (2007). Sensitization of TRAIL-resistant LNCaP cells by resveratrol (3, 4′, 5 tri-hydroxystilbene): Molecular mechanisms and therapeutic potential. J. Mol. Signal..

[69] Rosa L.D.S., Jordão N.A., Soares N.D., DeMesquita J.F., Monteiro M., Teodoro A.J. (2018). Pharmacokinetic, Antipro-liferative and Apoptotic Effects of Phenolic Acids in Human Colon Adenocarcinoma Cells Using In Vitro and In Silico Approaches. Molecules.

[70] Totta P., Acconcia F., Leone S., Cardillo I., Marino M. (2004). Mechanisms of Naringenin-induced Apoptotic Cascade in Cancer Cells: Involvement of Estrogen Receptor α and β Signalling. IUBMB Life.

[71] You B.R., Kim S.Z., Kim S.H., Park W.H. (2011). Gallic acid-induced lung cancer cell death is accompanied by ROS increase and glutathione depletion. Mol. Cell. Biochem..

[72] Scalbert A., Manach C., Morand C., Remesy C., Jimenez L. (2005). Dietary polyphenols and the prevention of diseases. Crit. Rev. Food Sci. Nutr..

[73] Thakur V.S., Gupta K., Gupta S. (2012). The chemopreventive and chemotherapeutic potentials of tea polyphenols. Curr. Pharm. Biotechnol..

[74] Pandey K.B., Rizvi S.I. (2009). Plant polyphenols as dietary antioxidants in human health and disease. Oxidative Med. Cell. Longev..

[75] Perrone L., Squillaro T., Napolitano F., Terracciano C., Sampaolo S., Melone M.A.B. (2019). The Autophagy Signaling Pathway: A Potential Multifunctional Therapeutic Target of Curcumin in Neurological and Neuromuscular Diseases. Nutrients.

[76] Beltz L.A., Bayer D.K., Moss A.L., Simet I.M. (2006). Mechanisms of cancer prevention by green and black tea polyphenols. Anti-Cancer Agents Med. Chem..

[77] Anand P., Kunnumakkara A.B., Sundaram C., Harikumar K.B., Tharakan S.T., Lai O.S., Sung B., Aggarwal B.B. (2008). Cancer is a preventable disease that requires major lifestyle changes. Pharm. Res..

[78] Stoll E.A., Horner P.J., Rostomily R.C. (2013). The impact of age on oncogenic potential: Tumor-initiating cells and the brain microenvironment. Aging Cell.

[79] Niedzwiecki A., Roomi M.W., Kalinovsky T., Rath M. (2016). Anticancer efficacy of polyphenols and their combinations. Nutrients.

[80] Shahcheraghi S.H., Zangui M., Lotfi M., Ghayour-Mobarhan M., Ghorbani A., Jaliani H.Z., Sadeghnia H.R., Sahebkar A. (2019). Therapeutic Potential of Curcumin in the Treatment of Glioblastoma Multiforme. Curr. Pharm. Des..

[81] Zhuang W., Long L., Zheng B., Ji W., Yang N., Zhang Q., Liang Z. (2012). Curcumin promotes differentiation of glioma-initiating cells by inducing autophagy. Cancer Sci..

[82] Wang X., Deng J., Yuan J., Tang X., Wang Y., Chen H., Liu Y., Zhou L. (2017). Curcumin exerts its tumor suppressive function via inhibition of NEDD4 oncoprotein in glioma cancer cells. Int. J. Oncol..

[83] Mukherjee S., Baidoo J., Fried A., Atwi D., Dolai S., Boockvar J., Symons M., Ruggieri R., Raja K., Banerjee P. (2016). Curcumin changes the polarity of tumor-associated microglia and eliminates glioblastoma. Int. J. Cancer.

[84] Lee J.E., Yoon S.S., Moon E.Y. (2019). Curcumin-Induced Autophagy Augments Its Antitumor Effect against A172 Human Glioblastoma Cells. Biomol. Ther..

[85] Maiti P., Scott J., Sengupta D., Al-Gharaibeh A., Dunbar G.L. (2019). Curcumin and Solid Lipid Curcumin Particles Induce Autophagy, but Inhibit Mitophagy and the PI3K-Akt/mTOR Pathway in Cultured Glioblastoma Cells. Int. J. Mol. Sci..

[86] Park K.S., Yoon S.Y., Park S.H., Hwang J.H. (2019). Anti-Migration and Anti-Invasion Effects of Curcumin via Suppression of Fascin Expression in Glioblastoma Cells. Brain Tumor. Res. Treat..

[87] Sak K. (2019). Radiosensitizing Potential of Curcumin in Different Cancer Models. Nutr. Cancer.

[88] Kielbik A., Wawryka P., Przystupski D., Rossowska J., Szewczyk A., Saczko J., Kulbacka J., Chwiłkowska A. (2019). Effects of Photosensitization of Curcumin in Human Glioblastoma Multiforme Cells. In Vivo.

[89] Pawlowska E., Szczepanska J., Szatkowska M., Blasiak J. (2018). An Interplay between Senescence, Apoptosis and Autophagy in Glioblastoma Multiforme-Role in Pathogenesis and Therapeutic Perspective. Int. J. Mol. Sci..

[90] Pistollato F., Bremer-Hoffmann S., Basso G., Cano S.S., Elio I., Vergara M.M., Giampieri F., Battino M. (2016). Targeting Glioblastoma with the Use of Phytocompounds and Nanoparticles. Target Oncol..

[91] Sato A., Okada M., Shibuya K., Watanabe E., Seino S., Suzuki K., Narita Y., Shibui S., Kayama T., Kitanaka C. (2013). Resveratrol promotes proteasome-dependent degradation of Nanog via p53 activation and induces differentiation of glioma stem cells. Stem. Cell Res..

[92] Xiong W., Yin A., Mao X., Zhang W., Huang H., Zhang X. (2016). Resveratrol suppresses human glioblastoma cell migration and invasion via activation of RhoA/ROCK signaling pathway. Oncol. Lett..

[93] Cilibrasi C., Riva G., Romano G., Cadamuro M., Bazzoni R., Butta V., Paoletta L., Dalprà L., Strazzabosco M., Lavitrano M. (2017). Resveratrol Impairs Glioma Stem Cells Proliferation and Motility by Modulating the Wnt Signaling Pathway. PLoS ONE.

[94] Yang Y.P., Chang Y.L., Huang P.I., Chiou G.Y., Tseng L.M., Chiou S.H., Chen M.H., Chen M.T., Shih Y.H., Chang C. (2012). Resveratrol suppresses tumorigenicity and enhances radiosensitivity in primary glioblastoma tumor initiating cells by inhibiting the STAT3 axis. J. Cell Physiol..

[95] Mirzazadeh A., Kheirollahi M., Farashahi E., Sadeghian-Nodoushan F., Sheikhha M.H., Aflatoonian B. (2017). Assessment Effects of Resveratrol on Human Telomerase Reverse Transcriptase Messenger Ribonucleic Acid Transcript in Human Glioblastoma. Adv. Biomed. Res..

[96] Song X., Shu X.H., Wu M.L., Zheng X., Jia B., Kong Q.Y., Liu J., Li H. (2018). Postoperative resveratrol administration improves prognosis of rat orthotopic glioblastomas. BMC Cancer.

[97] Song Y., Chen Y., Li Y., Lyu X., Cui J., Cheng Y., Zheng T., Zhao L., Zhao G. (2019). Resveratrol Suppresses Epithelial-Mesenchymal Transition in GBM by Regulating Smad-Dependent Signaling. Biomed. Res. Int..

[98] Öztürk Y., Günaydın C., Yalçın F., Nazıroğlu M., Braidy N. (2019). Resveratrol Enhances Apoptotic and Oxidant Effects of Paclitaxel through TRPM2 Channel Activation in DBTRG Glioblastoma Cells. Oxid. Med. Cell Longev..

[99] Yokoyama S., Hirano H., Wakimaru N., Sarker K.P., Kuratsu J. (2001). Inhibitory effect of epigallocatechin-gallate on brain tumor cell lines in vitro. Neuro-Oncology.

[100] McLaughlin N., Annabi B., Bouzeghrane M., Temme A., Bahary J.P., Moumdjian R., Beliveau R. (2006). The Survivin-mediated radio-resistant phenotype of glioblastomas is regulated by RhoA and inhibited by the green tea polyphenol (−)-epigallocatechin-3-gallate. Brain Res..

[101] Chen T.C., Wang W., Golden E.B., Thomas S., Sivakumar W., Hofman F.M., Louie S.G., Schönthal A.H. (2011). Green tea epigallocatechin gallate enhances therapeutic efficacy of temozolomide in orthotopic mouse glioblastoma models. Cancer Lett..

[102] Zhang Y., Wang S.X., Ma J.W., Li H.Y., Ye J.C., Xie S.M., Du B., Zhong X.Y. (2015). EGCG inhibits properties of glioma stem-like cells and synergizes with temozolomide through downregulation of P-glycoprotein inhibition. J. Neurooncol..

[103] Sui X.M., Wang J.X., Zhu Q.W., Zhang Q.F. (2016). Epigallocatechin-3-gallate induces apoptosis and proliferation inhibition of glioma cell through suppressing JAK2/STAT3 signaling pathway. Int. J. Clin. Exp. Med..

[104] Roomi M.W., Kalinovsky T., Rath M., Niedzwiecki A. (2017). Modulation of MMP-2 and MMP-9 secretion by cytokines, inducers and inhibitors in human glioblastoma T-98G cells. Oncol. Rep..

[105] Gurusinghe K.R.D.S.N.S., Mishra A., Mishra S. (2018). Glucose-regulated protein 78 substrate-binding domain alters its conformation upon EGCG inhibitor binding to nucleotide-binding domain: Molecular dynamics studies. Sci. Rep..

[106] Grube S., Ewald C., Kögler C., Lawson-McLean A., Kalff R., Walter J. (2018). Achievable Central Nervous System Concentrations of the Green Tea Catechin EGCG Induce Stress in Glioblastoma Cells in Vitro. Nutr. Cancer.

[107] Xie C.R., You C.G., Zhang N., Sheng H.S., Zheng X.S. (2018). Epigallocatechin Gallate Preferentially Inhibits O6-Methylguanine DNA-Methyltransferase Expression in Glioblastoma Cells Rather than in Nontumor Glial Cells. Nutr. Cancer.

[108] Udroiu I., Marinaccio J., Sgura A. (2019). Epigallocatechin-3-gallate induces telomere shortening and clastogenic damage in glioblastoma cells. Environ. Mol. Mutagen..

[109] Santos B.L., Silva A.R., Pitanga B.P., Sousa C.S., Grangeiro M.S., Fragomeni B.O., Coelho P.L., Oliveira M.N., Menezes-Filho N.J., Costa M.F. (2011). Antiproliferative, proapoptotic and morphogenic effects of the flavonoid rutin on human glioblastoma cells. Food Chem..

[110] Jakubowicz-Gil J., Langner E., Badziul D., Wertel I., Rzeski W. (2013). Apoptosis induction in human glioblastoma multiforme T98G cells upon temozolomide and quercetin treatment. Tumour Biol. J. Int. Soc. Oncodev. Biol. Med..

[111] Pozsgai E., Bellyei S., Cseh A., Boronkai A., Racz B., Szabo A., Sumegi B., Hocsak E. (2013). Quercetin increases the efficacy of glioblastoma treatment compared to standard chemoradiotherapy by the suppression of PI-3-kinase-Akt pathway. Nutr. Cancer.

[112] Zhang P., Sun S., Li N., Ho A.S.W., Kiang K.M.Y., Zhang X., Cheng Y.S., Poon M.W., Lee D., Pu J.K.S. (2017). Rutin increases the cytotoxicity of temozolomide in glioblastoma via autophagy inhibition. J. Neurooncol..

[113] Liu Y., Tang Z.G., Lin Y., Qu X.G., Lv W., Wang G.B., Li C.L. (2017). Effects of quercetin on proliferation and migration of human glioblastoma U251 cells. Biomed. Pharm..

[114] Liu Y., Tang Z.G., Yang J.Q., Zhou Y., Meng L.H., Wang H., Li C.L. (2017). Low concentration of quercetin antagonizes the invasion and angiogenesis of human glioblastoma U251 cells. Onco Targets Ther..

[115] Gentile M.T., Ciniglia C., Reccia M.G., Volpicelli F., Gatti M., Thellung S., Florio T., Melone M.A., Colucci-D’Amato L. (2015). Ruta graveolens L. induces death of glioblastoma cells and neural progenitors, but not of neurons, via ERK 1/2 and AKT activation. PLoS ONE.

[116] Taylor M.A., Khathayer F., Ray S.K. (2019). Quercetin and Sodium Butyrate Synergistically Increase Apoptosis in Rat C6 and Human T98G Glioblastoma Cells Through Inhibition of Autophagy. Neurochem. Res..

[117] Tora M.S., Xenos D., Texakalidis P., Boulis N.M. (2019). Treatment of neurofibromatosis 1-associated malignant peripheral nerve sheath tumors: A systematic review. Neurosurg. Rev..

[118] Angelo L.S., Wu J.Y., Meng F., Sun M., Kopetz S., McCutcheon I.E., Slopis J.M., Kurzrock R. (2011). Combining curcumin (diferuloylmethane) and heat shock protein inhibition for neurofibromatosis 2 treatment: Analysis of response and resistance pathways. Mol. Cancer.

[119] Blakeley J. (2012). Development of drug treatments for neurofibromatosis type 2-associated vestibular schwannoma. Curr. Opin. Otolaryngol. Head Neck Surg..

[120] Turrini E., Ferruzzi L., Fimognari C. (2015). Potential effects of pomegranate polyphenols in cancer prevention and therapy. Oxidative Med. Cell. Longev..

[121] Esposito T., Schettino C., Polverino P., Allocca S., Adelfi L., D’Amico A., Capaldo G., Varriale B., Di Salle A., Peluso G. (2017). Synergistic Interplay between Curcumin and Polyphenol-Rich Foods in the Mediterranean Diet: Therapeutic Prospects for Neurofibromatosis 1 Patients. Nutrients.

[122] Lee M.J., Tsai Y.J., Lin M.Y., You H.L., Kalyanam N., Ho C.T., Pan M.H. (2019). Calebin-A induced death of malignant peripheral nerve sheath tumor cells by activation of histone acetyltransferase. Phytomedicine.

[123] Hu M., Wu B., Liu Z. (2017). Bioavailability of polyphenols and flavonoids in the era of precision medicine. Mol Pharm.

[124] Teng H., Chen L. (2019). Polyphenols and bioavailability: An update. Crit. Rev. Food Sci. Nutr..

[125] Pandareesh M.D., Mythri R.B., Srinivas Bharath M.M. (2015). Bioavailability of dietary polyphenols: Factors contributing to their clinical application in CNS diseases. Neurochem. Int..

[126] Squillaro T., Peluso G., Melone M.A.B. (2017). Nanotechnology-based polyphenol delivery: A novel therapeutic strategy for the treatment of age-related neurodegenerative disorder. Austin Aging Res..

[127] Figueira I., Garcia G., Pimpão R.C., Terrasso A.P., Costa I., Almeida A.F., Tavares L., Pais T.F., Pinto P., Ventura M.R. (2017). Polyphenols journey through blood-brain barrier towards neuronal protection. Sci. Rep..

[128] Filosa S., Di Meo F., Crispi S. (2018). Polyphenols-gut microbiota interplay and brain neuromodulation. Neural. Regen. Res..

[129] Finicelli M., Squillaro T., Di Cristo F., Di Salle A., Melone M.A.B., Galderisi U., Peluso G. (2019). Metabolic syndrome, Mediterranean diet, and polyphenols: Evidence and perspectives. J. Cell Physiol..

[130] Squillaro T., Cimini A., Peluso G., Giordano A., Melone M.A.B. (2018). Nano-delivery systems for encapsulation of dietary polyphenols: An experimental approach for neurodegenerative diseases and brain tumors. Biochem. Pharm..

[131] Singh H. (2016). Nanotechnology Applications in Functional Foods; Opportunities and Challenges. Prev. Nutr. Food Sci..

